# Supplementary material for: Heterogeneous optoelectronic characteristics of Si micropillar arrays fabricated by metal-assisted chemical etching
Source: Sci Rep. 2020 Oct 1;10:16349. doi: 10.1038/s41598-020-73445-x (PMC7530667; doi:10.1038/s41598-020-73445-x)

**SUPPORTING INFORMATION**

Heterogeneous Optoelectronic Characteristics of Si Micropillar Arrays Fabricated by Metal-Assisted Chemical Etching

Yang Qian^1^, David J. Magginetti^2^, Seokmin Jeon^3^, Yohan Yoon^3, 4^, Tony L. Olsen^5^, Maoji Wang^6^, Jordan M. Gerton^6^, and Heayoung P. Yoon^1, 2^

^1^ Electrical and Computer Engineering, University of Utah, Salt Lake City, Utah 84112, USA

^2^ Materials Science and Engineering, University of Utah, Salt Lake City, Utah 84112, USA

^3^ US Naval Research Laboratory, Washington, DC 20375, USA

^4^ Materials Engineering, Korea Aerospace University, Goyang 412-791, South Korea

^5^ Utah Nanofab, University of Utah, Salt Lake City, Utah 84112, USA

^6^ Physics and Astronomy, University of Utah, Salt Lake City, Utah 84112, USA

**[Figure S1] SEM (Scanning Electron Microscopy) Imaging**

High-resolution SEM images of MACE-fabricated Si pillar arrays were collected with an immersion lens at 5 keV electron beam irradiation (beam current of 100 nA). The pillar edge shows a structural roughness/porosity on the scale of a few 100 nm (Figure S1 a, b). The sidewall features follow these edge structures (Figure S1 a), consistent with the sidewall topography observed with our AFM (atomic force microscopy) measurements in Figure 4 (main text). We note that our AFM images revealed the nanoscale porosity on the Si rough surface (< 10 nm porosity).


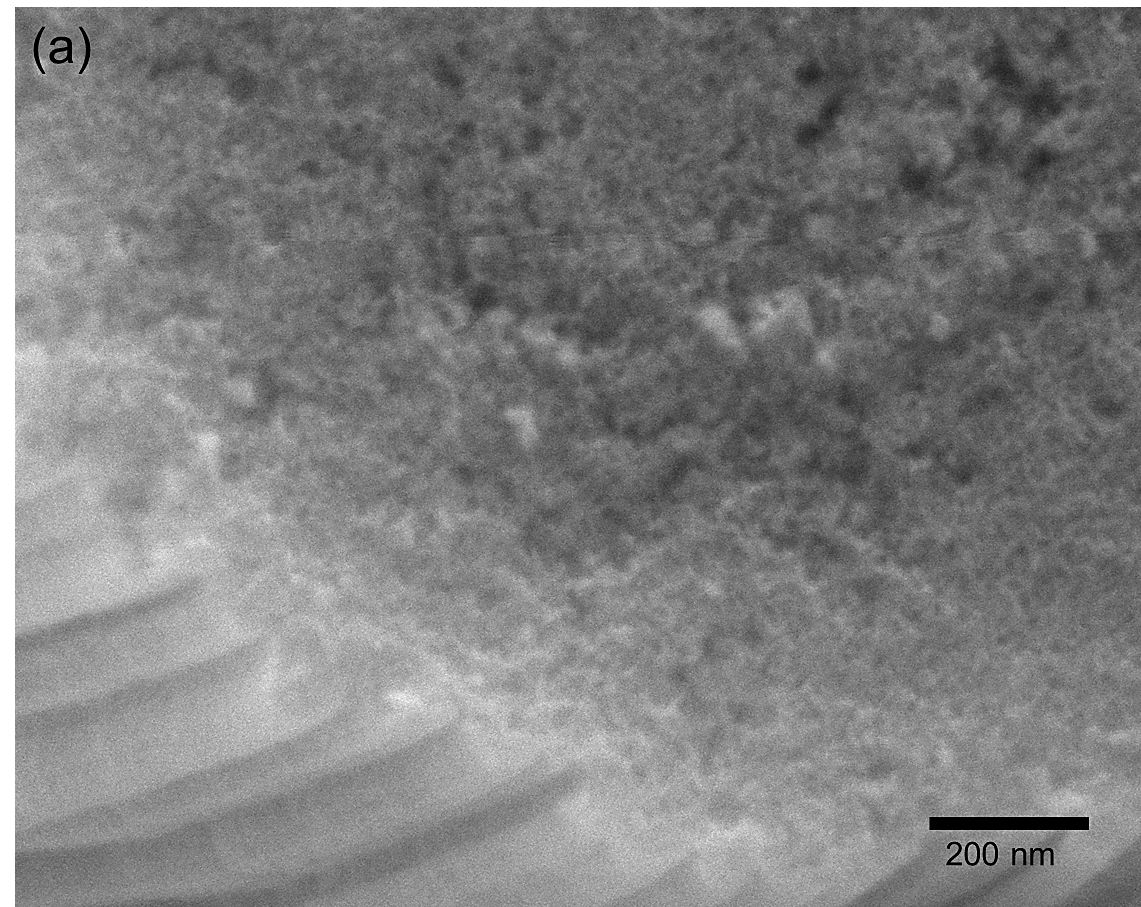


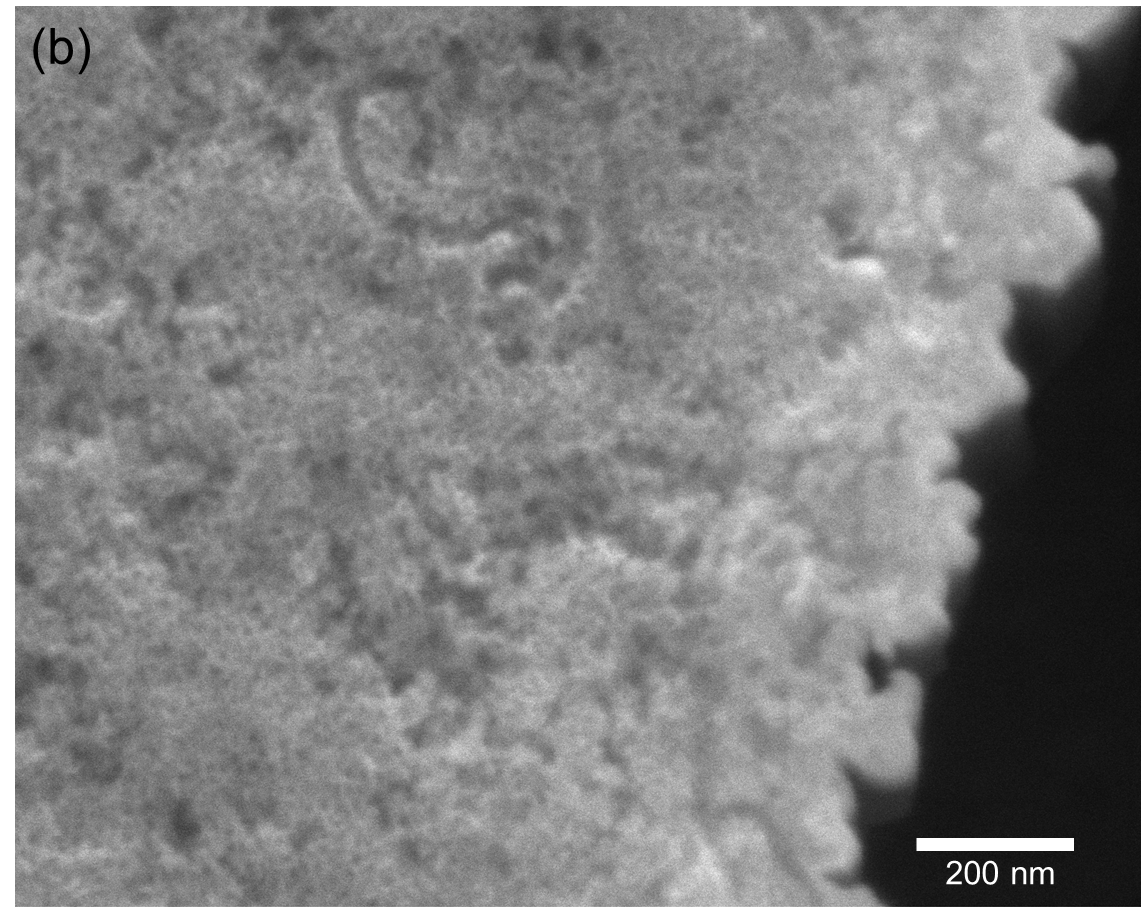


**[Figure S2] PL (Photoluminescence) Spectroscopy Analysis**

We used multiple Gaussian curve fit functions to decompose the PL peaks. The same curve fit was applied for eleven PL spectra in Figure 3e. Three peaks around 1.9 eV, 2.4 eV, and 2.7 eV were observed for all individual spectrum. The extracted data were plotted in Figure 3f and 3g.


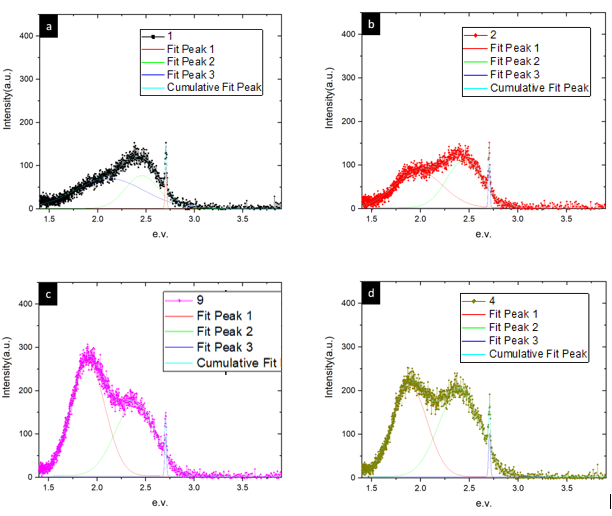


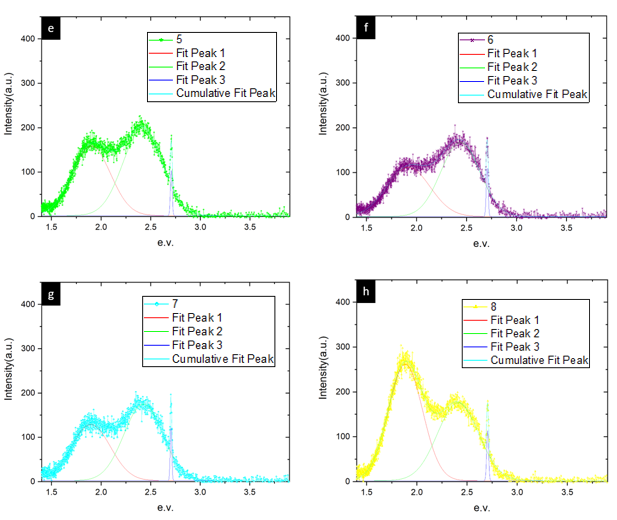


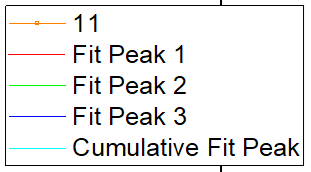


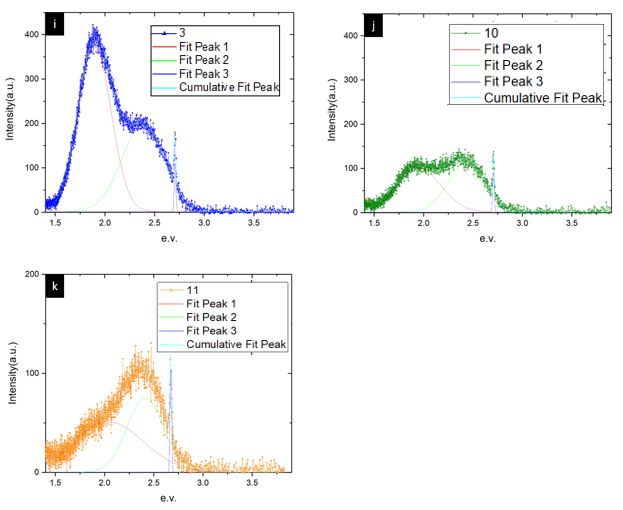

Supplement: Supplementary file 1 — Supplementary Information. [file 41598_2020_73445_MOESM1_ESM.docx]
